# Biomarker Landscape in Neuroendocrine Tumors With High-Grade Features: Current Knowledge and Future Perspective

**DOI:** 10.3389/fonc.2022.780716

**Published:** 2022-02-04

**Authors:** Michele Prisciandaro, Maria Antista, Alessandra Raimondi, Francesca Corti, Federica Morano, Giovanni Centonze, Giovanna Sabella, Alessandro Mangogna, Giovanni Randon, Filippo Pagani, Natalie Prinzi, Monica Niger, Salvatore Corallo, Erica Castiglioni di Caronno, Marco Massafra, Maria Di Bartolomeo, Filippo de Braud, Massimo Milione, Sara Pusceddu

**Affiliations:** ^1^ Medical Oncology Department, Fondazione IRCCS Istituto Nazionale dei Tumori, Milan, Italy; ^2^ Department of Oncology and Hemato-Oncology, University of Milan, Milan, Italy; ^3^ First Pathology Division, Department of Pathology and Laboratory Medicine, Fondazione IRCCS Istituto Nazionale dei Tumori, Milan, Italy; ^4^ Institute for Maternal and Child Health, IRCCS Burlo Garofalo, Trieste, Italy

**Keywords:** neuroendocrine tumors, neuroendocrine carcinoma (NEC), next-generation sequencing (NGS), PD-L1, high microsatellite instability (MSI-H)

## Abstract

Neuroendocrine tumors (NETs) are classified based on morphology and are graded based on their proliferation rate as either well-differentiated low-grade (G1) to intermediate (G2–G3) or poorly differentiated high-grade neuroendocrine carcinomas (NEC G3). Recently, in gastroenteropancreatic (GEP) NETs, a new subgroup of well-differentiated high-grade tumors (NET G3) has been divided from NEC by WHO due to its different clinical–pathologic features. Although several mutational analyses have been performed, a molecular classification of NET is an unmet need in particular for G3, which tends to be more aggressive and have less benefit to the available therapies. Specifically, new possible prognostic and, above all, predictive factors are highly awaited, giving the basis for new treatments. Alteration of *KRAS*, *TP53*, and *RB1* is mainly reported, but also druggable alterations, including *BRAF* and high microsatellite instability (MSI-H), have been documented in subsets of patients. In addition, PD-L1 demonstrated to be highly expressed in G3 NETs, probably becoming a new biomarker for G3 neuroendocrine neoplasm (NEN) discrimination and a predictive one for immunotherapy response. In this review, we describe the current knowledge available on a high-grade NET molecular landscape with a specific focus on those harboring potentially therapeutic targets in the advanced setting.

## Introduction

Neuroendocrine neoplasms (NENs) are a heterogeneous group of rare malignant cancers that arise from diffuse neuroendocrine cells. In recent years, the incidence and prevalence of NENs have steadily risen, with a 6.4-fold increase in age-adjusted incidence rate from 1.09 cases per 100,000 in 1973 to 6.98 per 100,000 in 2012 in the United States (1). About 62%–67% of all NEN cases are of gastroenteropancreatic (GEP) origin, 22%–27% of cases have a thoracic origin (lung and thymus NEN), and 10% of the primary tumor remains unknown (2–4). According to the 2019 WHO classification, GEP NENs are classified into well-differentiated neuroendocrine tumors (NETs) and poorly differentiated neuroendocrine carcinomas (NECs) based on both morphological features and proliferation rate (Ki-67 and/or mitotic index) (5). Recently, the NET category G3 was distinguished from the others. It is characterized by well-differentiated neoplasms but with a Ki-67 proliferative index >20%, which is typical of NECs. The need to recognize this new subgroup arose from the observation of a more favorable clinical trend and a different response to medical therapies of this subgroup of patients compared with patients with poorly differentiated tumors. Specifically, as we recently demonstrated, well-differentiated morphology constitutes an independent prognostic factor for GEP NEN with Ki-67 of between 20% and 55% (NET G3 and NEC with Ki-67 20%–55%), while the 55% cutoff of Ki-67 is an independent prognostic factor for poorly differentiated GEP NENs (6). Ki-67 of the neuroendocrine component appears to be the main prognostic factor also for mixed neuroendocrine non-NENs (MiNEN), and lung large cell NECs (LCNECs) (7, 8). Different from NETs, GEP NECs encompass poorly differentiated G3 neoplasms with Ki-67 proliferation index >20% and/or mitotic index >20 per 10 high-power fields (5). They are characterized by a proliferation of tumor cells with irregular nuclei and high mitotic features, with limited immunohistochemical staining for neuroendocrine markers, often displaying faint or focal staining for chromogranin A and diffuse synaptophysin expression (9). Of note, up to 40% of NECs may contain elements of non-neuroendocrine histology (9, 10). While well-differentiated NETs tend to have a relatively indolent behavior, with an excellent prognosis for NETs G1 (Ki-67 < 3%) and good to intermediate for NETs G2 (Ki-67 3–20%), NETs G3 and NECs display an aggressive disease course leading to poor survival outcomes with median overall survival (OS) ranging from 7.5 to 15 months (6, 11). NENs of the lung, on the other hand, according to the latest WHO classification of thoracic tumors (5th edition 2021), remain classified into four histological variants according to necrosis amount and mitotic count: typical and atypical carcinoid, LCNEC, and small cell lung cancer (SCLC) (12). According to the unifying nomenclature proposed by the International Agency for Research on Cancer (IARC) and the WHO Classification of Tumours Group, carcinoids are NETs with low mitoses number and absent or focal necrosis, contrary to LCNECs and SCLCs, which are NECs with extensive necrosis and high mitosis number. Therefore, high-grade NENs of the lung and thymus include SCLC and LCNECs by definition (12). Although several next-generation sequencing (NGS) analyses have been performed, one of the main unmet needs is the lack of a molecular classification of NETs, in particular for high-grade tumors, which tend to be more aggressive and have less benefit from the scantily available therapies. Chemotherapy with platinum compounds plus etoposide still represents the gold standard of first-line treatment, whereas the use of other chemotherapeutic agents [such as irinotecan, fluoropyrimidines, and temozolomide (TMZ)] in further lines of treatment is mostly supported by non-randomized or retrospective evidences (13). Nevertheless, recent progress in tumor genomic profiling has shed some light on the complex molecular scenario of high-grade NETs, identifying a wide range of genomic alterations (mutations, translocations, or amplifications) that could play both a prognostic role, conferring a much aggressive behavior to the tumor, and a predictive one, identifying tumors that may be suitable to biologic agents, allowing a deeper treatment personalization. In this review, we will describe all the available data on the landscape of molecular alteration in NENs with high-grade features (NETs G3 and NECs) particularly focusing on their future clinical and therapeutic role.

## Genomic Alterations

Personalized oncology, defined as the use of molecular profiling to drive treatment strategies for a single patient, is currently a reality in many cancers. In the last decades, the discovery of several oncogenic drive mutations in different malignancies, i.e., *Epidermal Growth Factor Receptor*, and *BRAF* mutations, led to the development of a huge number of targeted drugs with a totally different mechanism of action compared with chemotherapy, which is still, however, commonly used. As far as NENs are concerned, excluding well-established hereditary genetic syndromes caused by germline mutations and commonly associated with well-differentiated NETs, only a few data exist on tissue somatic gene alterations as markers of prognosis or predictive of treatment benefit in high-grade NETs. However, NGS data are expected to emerge rapidly in this field. In the first reports, all the genomic abnormalities observed seemed to be similar to those of the corresponding exocrine neoplasm of the same site (14, 15). Nonetheless, additional mutations specifically related to NETs were also described. Several studies showed that *TP53*, *Kirsten rat sarcoma* (*KRAS*), and *Retinoblastoma 1* (*RB1*) mutations were highly represented in NECs and represent markers of poor differentiation (16–21). On the contrary, several gene mutations may characterize well-differentiated NETs, as observed with *Menin 1* (*MEN1*), *Death Domain Associated Protein* (*DAXX*), and *alpha-thalassemia/mental retardation*, *X-linked* (*ATRX*) mutations in well-differentiated pancreatic NETs (22). Based on this, along with morphological differentiation and proliferation rate, NETs and NECs can be classified and differentiated according to their molecular profile (10). In GEP NETs, the presence of *TP53*, *KRAS*, and *RB1* mutations may also help in differentiating pancreatic NECs from NETs G3 and in predicting the response to platinum-based chemotherapy in the first ones (23). Molecular classification can be also hypothesized in lung NENs according to their genomic alterations (24). Mutations in *TP53* and *RB1* are present in all classes of lungs NENs (typical and atypical carcinoids, SCLCs, and LCNECs) but significantly enriched in NECs (24). Specifically, when mutations and copy number changes were combined, MEN1 alterations were almost exclusive to carcinoids, whereas alterations of the *TP53* and *RB1* cell cycle regulation genes and *Phosphatidylinositol-4,5-Bisphosphate 3-Kinase* (*PI3K*)/*AKT*/*Mechanistic Target of Rapamycin Kinase* (*mTOR*) pathway genes were significantly enriched in carcinomas (25). Recently, Simbolo et al., based on transcriptomic and genomic data, separated atypical carcinoids and LCNECs into three different and clinically relevant molecular diseases (26). Furthermore, in LCNECs, two mutually exclusive genomic subtypes have been identified: one profile shows concurrent *TP53* and *RB1* mutations similarly to SCLC, whereas the other subtype is predominantly RB1 wild-type and displays concurrent biallelic *TP53* and *Serine/Threonine Kinase 11 (STK11)/Kelch Like ECH Associated Protein 1 (KEAP1)* alterations, similarly to non-SCLC instead (27, 28). Besides a potentially new molecular classification, deep sequencing would be helpful also to predict patient outcomes. Indeed, *RB1* mutation and *Telomerase Reverse Transcriptase (TERT)* gain are shown to be independent unfavorable prognostic markers in all lung NENs, *MEN1* mutation was associated with poor prognosis in atypical carcinoids, and *Histone-Lysine N-Methyltransferase 2D* mutation was associated with longer survival in SCLCs (25, 26). Likewise, to those genes described before, chromatin-modifying genes, in particular, *AT-Rich Interaction Domain 1A (ARID1A)*, could also play a major role in atypical carcinoids and LCNECs (24, 25).

In addition to those previously described, mutations of other genes have been also described in NECs (**Table 1**) (16, 29). In a recent NGS dataset analysis, Chen *et al.* found that about 20.8% of patients with colorectal NECs harbored *BRAF* V600E mutation (20). This may represent a potential target for tyrosine kinase inhibitors (TKIs), such as dabrafenib and trametinib, as it happens in colorectal and lung cancers (29, 30, 103). Another novel potential therapeutic target is *Delta-like protein 3* (DLL3) (31), an inhibitory ligand of the Notch receptor pathway, which is highly expressed in lung NECs (about 80% of SCLCs and 65% of LCNECs) (32, 33), GEP NECs (34), and renal NECs (35). In a recent retrospective analysis, Liverani et al. demonstrated that Dll3 expression assessed *via* immunohistochemistry (IHC) was present in GEP NEC and absent in GEP NET G3, representing a valuable histological marker, for the diagnosis of NECs. In addition, Dll3 expression was also correlated with *RB1*-loss (p < 0.001), negative ^68^Ga-PET/CT scan (p = 0.001), and a worse OS (34). A correlation between Dll3 expression and *RB1*-loss was also observed in SCLC but not in LCNEC (27, 31). Dll3 has been recently studied as a potential target for a novel antibody-drug conjugate called rovalpituzumab tesirine. Despite early-phase trials showing encouraging single-agent antitumor activity, rovalpituzumab tesirine failed, unfortunately, to demonstrate OS superiority in SCLC over placebo as maintenance after platinum-based therapy (36) and over topotecan in second-line setting (37) in phase III trials. Nonetheless, there were several trials investigating the role of novel Dll3 inhibitors in SCLCs, LCNECs, and NECs (**Table 2**).

**Table 1:** Potential novel biomarkers in high-grade NET and relative therapeutic agent.

Molecular Target	Disease	Clinical Correlations	Targeted Therapies	Ref
**TP53**	NECs	Marker of poor differentiation	None	(10, 16–21, 23–28)
**KRAS**	NECs	Marker of poor differentiation	None	(10, 16–21, 23)
**RB1**	NECs	Marker of poor differentiation	None	(10, 16–21, 23–29)
	Lung NETs	Worse prognosis		
**MEN1**	GEP NETs	Marker of well differentiation	None	(22, 24–28)
	Carcinoids	Diagnostic marker		
		Worse prognosis (AC)		
**DAXX**	GEP NETs	Marker of well differentiation	None	(22)
**ATRX**	GEP NETs	Marker of well differentiation	None	(22)
**ARID1A**	Lung NETs	Pathogenetic role	None	(24, 25)
		Enhancing mutational burden		
**BRAF**	Colorectal NECs	Response to BRAF-MEK inhibition	BRAF-MEK inhibitors	(20–30)
**DLL3**	GEP NECs	Marker of poor differentiation	Rovalpituzumab tesirine	(27, 31–37)
		Negative 68Ga-PET		
		Worse prognosis		
	SCLC	Correlated to RB1-loss		
	LCNEC	None		
	Renal NECs	None		
**BRCA**	Pancreatic NETs	Response to platinum-based regimes	PARP inhibitors	(22, 38–41)
	Prostatic NECs	Response to PARP inhibitors		
**SLFN11**	SCLC	Response to platinum-based regimes	PARP inhibitors	(42–45)
		Response to PARP inhibitors		
**ALK**	SCLCLCNEC	Worse prognosis	ALK inhibitors	(46–55)
**NTRK**	GEP NECs	Response to NTRK inhibitors	Entrectinib, larotrectinib, taletrectinib	(56–61)
	SCLC			
	LCNEC			
**PD-L1**	GEP NECs	Marker of poor differentiation	Immune checkpoint inhibitors	(16, 62–70)
		Worse prognosis		
		Response to immunotherapy		
	SCLC	Response to immunotherapy		
	LCNEC	Response to immunotherapy		
**H-MSI**	Gastric/colorectal NECs	Response to immunotherapy	Immune checkpoint inhibitors	(71–75)
**TMB**	GEP NECs	Response to immunotherapy	Immune checkpoint inhibitors	(21, 76–84)
	SCLC			
	LCNEC			
**miRNAs**	GEP NETs	Diagnostic markers	None	(85–102)
	Lung NETs	Prognostic markers		

NEC, neuroendocrine carcinoma; GEP, gastroenteropancreatic; NETs, neuroendocrine tumors; SCLC, small cell lung cancer; LCNEC, large cell neuroendocrine carcinoma; AC, atypical carcinoid; KRAS, Kirsten rat sarcoma; RB1, Retinoblastoma 1; MEN1, Menin 1; DAXX, Death Domain Associated Protein; ATRX, alpha-thalassemia/mental retardation, X-linked; ARID1A, AT-Rich Interaction Domain 1A; DLL3, Delta-like protein 3; PARP, Poly[ADP-ribose] polymerase 1; SLFN11, Schlafen 11; ALK, Anaplastic Lymphoma Kinase; NTRK, Neurotrophic receptor tyrosine kinase; PD-L1, programmed cell death protein ligand 1; H-MSI, high microsatellite instability; TMB, tumor mutational burden; miRNA, microRNA.

**Table 2:** Ongoing molecular-driven clinical trial involving high-grade NETs.

Drug(s)	Target	NCT Number	Patient Population	Phase
**Pembrolizumab**	H-MSI	NCT02628067	Solid tumors including NETs	II
**INCB099318**	H-MSI (cohort 2)	NCT04272034	Solid tumors including NETs	I
**BI 764532**	DLL3	NCT04429087	SCLC, LCNEC, NEC	Ib
**Entrectinib**	NTRK 1, 2, 3/ALK/ROS1	NCT02568267	Solid tumors including NETs	II
**Pralsetinib (BLU-667)**	RET	NCT03037385	Solid tumors including NETs	Ib/II
**Selpercatinib (LOXO-292)**	RET	NCT03157128	Solid tumors including NETs	I/II
**Encorafenib + binimetinib**	BRAF V600	NCT03864042	Solid tumors including NETs	I
**Avapritinib**	CKIT/PDGFRA	NCT04771520	Solid tumors including NETs	II

Data taken from clinicalTrials.com.

H-MSI, high microsatellite instability; DLL3, Delta-like protein 3; NTRK, Neurotrophic receptor tyrosine kinase; ALK, Anaplastic Lymphoma Kinase; NETs, neuroendocrine tumors; SCLC, small cell lung cancer; NEC, neuroendocrine carcinoma; LCNEC, large cell neuroendocrine carcinoma.

Furthermore, the role of the homologous recombination repair of the double-stranded DNA pathway in the pathogenesis of NENs has been also recently suggested (42). Recent studies have shown, indeed, that pancreatic NENs can be associated with germline pathogenic variants in genes involved in DNA damage repair, such as *MutY DNA Glycosylase*, *Checkpoint Kinase 2*, and above all *BRCA* (22, 38, 39). Two case reports described patients with prostate NEC, a highly aggressive histologic subtype of prostate cancer, one with germline and the second with somatic *BRCA* mutation, confirming platinum and *Poly[ADP-ribose] polymerase 1* (*PARP*) inhibitor sensibility similar to that of malignancies that frequently present this type of alteration (40, 41). Interestingly, in one of these cases, a novel reversion mutation that restores Brca 1/2 function was described, which might be the reason for primary resistance to *PAPR* inhibitors (41). In addition, the role of *Schlafen* (*SLFN*) 11 was also recently explored in SCLC. Besides its known antiviral properties, several preclinical and clinical studies have been shown its ability to sensitize cancer cells to DNA damaging agents such as chemotherapy and *PARP* inhibitors (42–45). In the MA 11.07 trial, 100 SCLC patients with 1–2 prior lines of therapy were treated with TMZ with either veliparib or placebo. Although the primary endpoint was not met in this trial, patients receiving the combination of TMZ plus veliparib had an almost 3-fold higher response rate as compared with the temozolomide plus placebo arm (39% vs. 19%). Median OS was 8.2 months in the temozolomide plus veliparib arm and 7.0 months in the temozolomide plus placebo arm (p = 0.50). However, a significantly longer progression-free survival (PFS) and OS were observed in patients receiving TMZ/veliparib combination who had detectable Slfn11 by IHC (44).

## Gene Rearrangements

The advances in the genomic profiling of solid tumors shed a light on the contribution of gene translocations, fusions, and amplifications in cancer initiation and progression. In addition to this, recently, gene rearrangements demonstrated also their potential role as prognostic and predictive markers or, most important, as therapeutic targets with the aim of personalizing the treatment algorithm (104). Nevertheless, the frequency of likely oncogenic recurrent gene fusions across the different cancer types is globally low, about 2%–3%, thus limiting the investigation on the singular genomic alterations (105, 106). In the setting of high-grade NENs, the deeper understanding of the molecular scenario recently provided interesting insights into their genomic landscape. With the limitation of the high clinical and molecular heterogeneity of NET G3/NEC, concerning gene fusions or amplifications, a few potential targets have been identified, with frequent tissue-specific features, and are under study (22, 107–110).

### Anaplastic Lymphoma Kinase

Anaplastic lymphoma kinase (*ALK*) gene encodes for the Alk protein, which is a receptor tyrosine kinase belonging to the insulin receptor superfamily that activates a downstream signaling pathway involved in cell survival, proliferation, and oncogenesis. A gene rearrangement involving the fusion of *ALK* with another gene, generating a novel driver oncogene, was first identified in anaplastic large cell lymphoma and afterward in other tumors, i.e., lung cancer (in about 5% of cases), and it represents nowadays a key biomarker for targeted treatments, with a much improved clinical outcome (46, 111). In the setting of lung NENs, including typical and atypical carcinoids, SCLCs, and LCNECs, the occurrence of *ALK* fusions is extremely rare, with few cases reported (**Table 1**) (47–52). With the available literature data, the incidence of *ALK* fusions in high-grade lung NENs appeared lower than in NSCLC, <3% versus 3%–5%. In a dataset of 108 patients with lung NENs, *ALK* fusions were reported in 0.9% of cases (53). In these cases, no associations with a particular histological type were observed, and the main fusion partner was *EMAP Like 4*, as in NSCLC. Rarer partners have been reported, such as *Kinesin Family Member 5B* with no impact on the clinical and therapeutic outcomes (48, 54). Interestingly, most NENs with *ALK* translocation were characterized by high-grade and advanced stage with disseminated lesions, even to the brain, with features that closely correlate with a poor prognosis. Therefore, the rearrangement of *ALK* in lung NEC may represent a specific molecular subtype endowed with more aggressive behavior (47). The diagnostic assessment should include either fluorescence *in situ* hybridization (FISH), reverse transcription PCR, or NGS to confirm the evidence of Alk expression by IHC, especially in cases with focal or heterogeneous expression. In fact, in a high-sensitivity Alk immunostaining on 227 lung NEC tissue microarrays dataset, it was shown that focal positivity with heterogeneous intensity did not correlate with *ALK* rearrangement/amplification in FISH or somatic mutation. Therefore, the aberrant expression of Alk could represent a potential pitfall in the molecular diagnosis of lung NECs, and its relevance relies particularly on the potential therapeutic implication of targeted treatment with Alk inhibitors (55). Due to their practice-changing results on NSCLC, crizotinib, ceritinib, and alectinib were investigated also in lung NECs harboring *ALK* fusion, showing significant disease responses with manageable tolerability in several cases (about 7 partial responses on the 13 cases collected in a literature-based case series review) (47, 49, 51, 54). Nevertheless, the low level of evidence, due to the rarity of the disease and the low frequency of this alteration, limits the clinical implication of *ALK* rearrangement in lung NECs. The greatest burden of data on *ALK* fusions has been collected for lung NECs, given the relevant role in the therapeutic management of NSCLC patients, whereas for non-lung NECs, the evidence of *ALK* fusions/amplifications is scarce, with reports of complete lack of expression in pancreatic NETs (0/46 cases) (46).

### Neurotrophic Receptor Tyrosine Kinase

The neurotrophic receptor tyrosine kinase (*NTRK*) is a tyrosine kinase receptor family including NTRK 1, 2, and 3, which encode the tropomyosin receptor kinase receptors, Trka, Trkb, and Trkc, respectively, involved in normal development, survival, and functionality of the nervous system. The Trk receptor, thanks to the binding with its ligand, homodimerizes and activates a downstream signaling cascade that modulates the activity of several key pathways including *RAS/MAPK* and *mTOR/AKT*. In solid tumors, *NTRK* translocations may occur, resulting in constitutively active protein fusions that display an oncogenic action (112). *NTRK* fusions are a rare finding in the most frequent tumors, although they are enriched in selected low-frequency cancers, such as secretory breast carcinoma, mammary analog secretory carcinoma, and congenital infantile fibrosarcoma, where *NTRK* fusion represents a defining diagnostic parameter (113, 114). In a large dataset of 2,417 NET patients, a total number of 6 cases (0.3%) of *NTRK* fusions were identified, including both intra- and inter-chromosomal translocations, and frequent or unique fusion partners, with no specific characteristics for organ of origin (lung, pancreas, uterus, and unknown primary) although with a peculiar selection for high-grade tumors as NECs or LCNECs (56). The relevance of *NTRK* fusions, aside from their low prevalence in solid tumors, is the potential therapeutic implication since *NTRK* rearrangements have emerged as a powerful actionable driver for targeted therapy. Recently, the selective inhibitors entrectinib and larotrectinib showed practice-changing results in the treatment of tumors with *NTRK* fusions, leading to the agnostic approval of the Food and Drug Administration (FDA) in advanced adult or pediatric tumors bearing this alteration (57–59). For NETs, the evidence collected on this topic is limited. In detail, a patient with metastatic well-differentiated NET, likely originating from the small intestine, bearing an ETS Variant Transcription Factor 6-NTRK3 fusion, was treated with entrectinib in the STARTRK2 trial with a rapid and meaningful tumor response preceded by initial tumor growth and necrosis (60). Moreover, 12 patients with NENs were treated with taletrectinib, a *ROS1/NTRK* inhibitor in a phase I study, reporting 1 partial response and 7 stable diseases according to Response Evaluation Criteria in Solid Tumors (RECIST) criteria, with a manageable toxicity profile (61). Although limited, these results appear promising for further investigations besides being impaired by the double rarity of the cases, that is, NETs that represent rare cancers and NTRK fusions that are a low-frequency molecular alteration (**Table 1**).

### Human Epidermal Growth Factor Receptor 2

*Human epidermal growth factor receptor 2* (*HER2* or *ERBB2*) is a member of the epidermal growth factor receptor family, involved in the regulation of tumor cell proliferation, apoptosis, adhesion, migration, and differentiation (115). *HER2* plays a central role in several tumors with evidence of amplification or overexpression in 7%–34% of all cancers, namely, breast, colon, bladder, ovarian, endometrial, lung, uterine cervix, head and neck, esophageal, and gastric cancers (116). It also represents a key target for the definition of the therapeutic algorithm in many cancer diseases, with numerous approved targeted agents that are able to provide a significant advantage on the clinical outcome (117, 118).

In NENs, the prognostic and predictive role of the amplification/overexpression of *HER2* has not been defined due to its rarity. Most data have been provided in NECs of breast and gastric primitivity, in concordance with the non-NENs (**Table 1**). In particular, breast NEC is a rare subset of breast cancer, accounting for 2%–5% of cases, even though neuroendocrine differentiation is observed in up to 20% of breast tumors, and it belongs mainly to the luminal subtype, with a low rate of Her2 positivity (119). The real impact of the amplification/overexpression of *HER2* on the prognosis of breast NENs is not clear, but an anti-Her2-targeted approach could be considered, even though solid evidence has not been collected (120). Concerning gastric cancer, case series studies have been performed on this topic, 51 gastric NECs (15 pure and 36 associated with adenocarcinoma and/or dysplasia) were analyzed, and *HER2* amplification was reported in 3 NECs (6%) and 7 (19%) mixed tumors. However, none of them displayed Her2 expression in IHC (121). Consistently, in the other three studies, Her2 expression in IHC was found to be negative, or *HER2* copy number analysis did not show amplification in 31 primitive gastric NECs overall (122–124). Therefore, the available evidence suggests that *HER2* may not represent a valid therapeutic target, although this could be influenced by intratumoral heterogeneity, and further studies should be warranted on this topic. Finally, a study encompassing an expression profiling analysis in LCNECs reported that two cases displayed overexpression of Her2 at IHC, suggesting a potential role as a treatment target to be further investigated (125).

## Immune Response Biomarkers

Recently, the introduction of immunotherapy dramatically changed the natural history of several cancer subtypes, like melanoma, lung cancer, and kidney cancer. Nonetheless, in some cases, the benefit of this treatment is confined only to a small portion of patients who show predictive biomarkers such as programmed cell death protein 1/ligand 1 (PD-1/PD-L1) or deficient mismatch repair (dMMR)/high microsatellite instability (MSI-H) status. In NENs, an increasing number of clinical trials with immunotherapy have been conducted (62). In March 2017, based on the results of the JAVELIN Merkel 200 trials, avelumab became the first FDA-approved agent for the treatment of metastatic Merkel cell carcinoma, a rare but aggressive NEC of the skin, and represented a new therapeutic option to improve patients’ survival (126, 127). Two years later, following the results of the IMpower133 trial, atezolizumab combined with chemotherapy was approved by the FDA for first-line treatment of extensive-stage SCLC. In this trial, the combination of chemotherapy and immunotherapy improved PFS and OS, with median PFS 5.2 versus 4.3 months (hazard ratio [HR] 0.77; 95% CI: 0.62–0.96; p = 0.02) and median OS 12.3 versus 10.3 months (HR 0.70; 95% CI: 0.54–0.91; p = 0.007), compared with chemotherapy alone (128). More recently, in phase II studies, the significant activity of spartalizumab in thoracic NENs (129) and also with the combination of ipilimumab plus nivolumab (objective response rate (ORR) 44%) in patients with non-pancreatic high-grade NENs (130).

With the exception of these few cases, unfortunately, there is a relatively low efficacy of immunotherapy in the unselected population of NENs, especially in GEP-NET. Therefore, one of the biggest challenges is to find those biomarkers that will allow to select those patients who will have a higher probability to benefit from this kind of treatment. Due to the heterogeneity of NENs and their rarity, as well as the fact that different primary tumor sites have different microenvironments, exploration in this field is indeed quite difficult. However, there is increasing evidence of the role that PD-1/PD-L1, tumor mutational burden (TMB), and dMMR/H-MSI status may also have in NENs (**Table 1**).

### Targeting PD-1/PDL-1 Pathway

PD-L1, an immune inhibitory protein, is often upregulated in tumor cells by interferon-gamma secreted from effector T cells when tumor antigens are recognized. By interacting with PD-1, PD-L1 can suppress many immune cell functions, especially T-cell activation favoring tumor cell immune escape. Expression levels of PD-L1 assessment *via* IHC on tumor cells are one of the predictive factors for patients treated with immunotherapy. Several retrospective studies demonstrated that PD-L1 expression is a frequent occurrence in high-grade GEP-NENs (62). Kim et al. firstly reported a 21.9% (7/32) PD-L1 expression rate in patients with metastatic GEP-NET, which was significantly associated (p = 0.008) with high-grade classification (63). Similar to this, PD-L1 positivity was found by Cavalcanti et al. in approximately 28% (16/57) of cases, and again, PD-L1 expression in both tumor and infiltrating immune cells was significantly higher in poorly differentiated NENs (p = 0.001), and its expression rates increased with the tumor aggressiveness. These findings may be related to possibly acquired resistance to immune surveillance by the upregulation of PD-L1 and the inhibition of peritumoral and intratumoral infiltrating lymphocytes limiting T cell-mediated tumor aggression (64). This may explain the higher PD-L1 expression rates observed in later case series restricted to high-grade GEP NETs. PD-L1 positivity of 48.8% was observed by Yang et al. in 43 gastric NECs (65), while 24.1% was described by Busico et al. in tumor-infiltrating lymphocytes (TILs) of 54 GEP high-grade NENs (16). In both studies, the high expression of PD-L1 was associated with poor OS. An increase of PD-L1 expression along the GEP-NENs grading stages was also reported in a retrospective study performed in our institution (66). In addition, we demonstrated that the transition from G1/G2 NETs to G3 NETs and G3 NECs is associated with profound changes in the tumor and stromal profile for inflammatory and immune-related markers and point to more frequent activation of adaptive immunity in NECs and a strong immune escape mechanism. Moreover, a subset of NECs has microenvironment features consistent with spontaneous activation of adaptive immunity (co-expression of CD3, CD4, CD8, PD-1, and PD-L1). Recently, we further evaluated the tumor microenvironment of high-grade NENs, by expanding the immune profiling to myeloid markers and identifying two prognostic subpopulations of tumors likely compatible with the “hot/cold tumor” idea: high-grade NENs characterized by a prevalent immune infiltrate cells had better survival (67). According to this, it was suggested that microenvironment-related immune and inflammatory markers can improve prognostic prediction in GEP-NENs when combined with the known prognostic factors, and they may predict potential responsiveness to immunotherapy of GEP NECs (66, 67). Furthermore, Bosch et al. demonstrated that high TILs and PD-1 expression are significantly associated with shorter survival and higher grading in GEP NENs. In addition, high expression of PD-L1 in tumor cells was associated with high rates of PD-1-positive lymphocytes and a significantly higher number of TILs. According to this, the authors suggested that in high TIL tumors, a higher number of PD-1-positive lymphocytes is present; thereby, tumor cells with the higher PD-L1 expression may be more able to escape from the immune response by upregulation of this pathway (68).

In summary, according to previous data, PD-L1 expression may be a useful biomarker first to discriminate GEP high-grade NENs, and then, it may potentially be a prognostic and, above all, predictive biomarker for response to immune checkpoint inhibitors (ICIs).

When considering high-grade lung NENs, PD-L1 positive rates tend to vary immensely across different studies. A reason for this wide range may be related to the use of different clones of anti-PD-L1 antibody for IHC along with variable cutoffs. But in those studies in which FDA-approved anti-PD-L1 antibodies and their relative cutoffs were used, expression rates tend to be low (69, 70). Interestingly, substantial PD-L1 expression occurs on stroma cells, including TILs, in SCLCs with favorable clinical outcomes. Overall, this relatively low PD-L1 expression along with the deficient expression of major histocompatibility complex class I molecules, which prevents tumor cells from presenting neoantigens to CD8+ T cells in the lymph nodes and inhibiting cytotoxic T lymphocytes, may be one of the main reasons why the efficacy of ICIs in SCLCs is not as good as that in NSCLCs (69).

### High Microsatellite Instability

H-MSI phenotype is another well-known biomarker that is under investigation in many neoplastic diseases. MMR proteins represent a complex system involved in DNA repair mechanisms, which ensure genomic integrity and remove DNA errors. Deficiency in MMR proteins (MLH1, MSH2, MSH6, and PMS2), commonly assessed by IHC, leads to an accumulation of DNA replication errors and mutations as well as expansion or contraction of microsatellite regions (131). The resulting hyper-mutated phenotype strongly enhances the formation of neo-antigens, making cancer cells more recognizable by the host immune system. Additionally, dMMR/H-MSI tumors have prominent lymphocyte infiltrates (132) and are more likely to express PD-L1 (133), which may predict response and durable clinical benefit to PD-1 blockade. For all these reasons, dMMR/H-MSI tumors are responsive to immunotherapy. Recently, FDA approval was granted for use of the anti-PD-1 antibody pembrolizumab for the treatment of metastatic non-hematologic cancers that are characterized by this alteration. Usually, dMMR is related to Lynch syndrome, which is caused by germline mutations of MMR proteins, leading to a 50%–70% lifetime risk of colorectal cancer, 40%–60% risk of endometrial cancer, and increased risks of several other malignancies (134). Despite this, dMMR/H-MSI can be also observed in sporadic cancer. Data on H-MSI in NENs are limited. Recent studies demonstrated that the presence of H-MSI phenotype on subsets of gastrointestinal (GI) NECs and MiNEN of the stomach and colorectum with an incidence rate up to 15%; it was mostly subsequent to MHL1 promoter methylation and with a more favorable prognosis (71, 72). In contrast, defects in DNA MMR proteins are rare in pancreatic NETs, small intestinal NETs (73, 74), and NECs of the endometrium (75) and cervix (75). These data suggest the prevalence of H-MSI in relatively low NETs; it is site-dependent and closely related to those organ sites in which H-MSI status is usually observed in the exocrine neoplastic counterparts, such as colorectal, gastric, and endometrial adenocarcinomas. Nevertheless, given the potential prognostic role and the clinical benefit of immunotherapy, dMMR/H-MSI testing must be encouraged as well as testing of other malignancies like colorectal cancer.

### Tumor Mutational Burden

In addition to the previous two TMBs is another recently discovered biomarker. It is broadly defined as the number of somatic mutations per megabase of interrogated genomic sequence. TMB is believed to be a key driver in the generation of immunogenic neopeptides displayed on major histocompatibility complexes on the tumor cell surface that influences patient response to ICIs (76). In a phase II study in patients with previously treated, unresectable, or metastatic solid tumors (KEYNOTE-158), TMB-high status (≥10 mut/Mb) was associated with a clinically meaningful improvement in the efficacy of pembrolizumab (77). According to this, the FDA approved pembrolizumab monotherapy for the subgroup of solid-tumor patients with TMB ≥10 mut/Mb who are treatment-refractory and lack satisfactory alternative treatment options.

TMB of NETs has not been fully studied yet. In a study of 4,125 patients with various GI cancer types, TMB levels have been analyzed. Among those, pancreatic NETs were found to have one of the lowest TMB (5.8 mut/Mb) (78). More recently, in another retrospective study, Shao et al. assessed TMB in 2,559 patients with different tumors. SCLC was found to have the highest median TMB (8.6 mut/Mb) and the highest rate of TMB-high (cutoff ≥10 mut/Mb, 40%), which is, interestingly, followed by the NETs (29.3%). However, this remarkable rate was driven by the patients with LCNEC in which TMB high rate was 45.6%. On the contrary, in the small bowel, colon, and rectal NETs grouped with LCNECs, the rate was lower (5.9%, 11.8%, and 0%, respectively). Despite this, no differences in OS were seen between TMB high and low tumors (79). High TMB and elevated TMB-high rates in SCLC were described in several other studies (80–83). Furthermore, the role of TMB as a predictive biomarker in extensive-stage SCLC was also explored in patients who were treated with nivolumab alone or combined with ipilimumab after the failure of at least one prior chemotherapy regimen (CheckMate032 trial). In these populations, ORR by treatment arm increased in patients whose tumors showed high versus medium versus low TMB levels. In addition, in patients with high TMB tumors, dual ICI treatment was associated with an impressive ORR of 46.2% and an estimated 1-year OS rate of 62.4% (84). Lastly, in another recent report, Hoffman-Censits et al. demonstrated that over 26% of small cell bladder cancer had high TMB, in particular TMB > 10 mutations/Mb, and 3% had TMB > 20 mut/Mb, with a median of 6.2 mut/Mb (21).

## MicroRNA

MicroRNAs (miRNAs) are small, non-coding RNAs with a length of 21–25 nucleotides and participate in gene regulation on the post-transcriptional level (135, 136). The role of miRNAs in cancerogenesis is now well-established, and several studies demonstrated the correlation between specific miRNA and different cancer subtypes (85). According to this, miRNA expression profiles are potentially exploited as practical supportive markers for differential NEN diagnosis and prognosis and provide adequate information on proper patient care and management (85–90). When considering pancreatic NETs, the expression of specific miRNAs is able to discriminate them from normal pancreas and other pathologic conditions such as pancreatic ductal adenocarcinoma and acinar pancreatic tumors (87, 91, 92). Specifically, the expressions of miR-144/451 cluster, miRNA-21, and MiR-193b were observed in insulinomas compared with normal pancreatic tissue, while miR-103 and miR-107 overexpression and miR-155 underexpression distinguish pancreatic NETs from acinar cell carcinomas (87, 91, 93). In addition to this, different miRNA expressions discriminate different clinical behaviors and prognoses of pancreatic NETs (88). Indeed, the overexpression of miR-21, miR-642, and miR-196a was found to be positively correlated with the Ki-67 proliferation index, whereas miR-210 correlated with the presence of liver metastases (93, 94). Additionally, miR-196a expression was significantly associated with stage, mitotic count, and decreased OS and disease-free survival (95). The pattern of miRNA expression was also explored in small bowel NETs (91, 96). MiR-7-5p, miR-182, miR-183, and miR-96-5p were found to be upregulated in NETs of the small bowel compared with normal tissue (91). In addition, the last three, along with the downregulation of miR-129-5p and miR-133a, were found to be overexpressed in the metastatic lesions compared with primary tumors (91, 97). Considering the prognostic role, high levels of circulating miR-21-5p and miR-22-3p and low levels of miR-150-5p were associated with shorter OS (98). Specific miRNA expressions were also reported in other GEP-NENs such as gastrin-induced miR-222 overexpression in hypergastrinemic patients and type 1 gastric NETs, which may be associated with tumor development by decreasing p27 expression (99); low levels of miR-96 and high levels of miR-133a expression in appendiceal carcinoids (91); underexpression of miR-186 in colorectal NETs (100)**;** and overexpression of miR-885-5p in rectal carcinoids (101). Lastly, in a recent study, Cavalcanti et al. reported that 8 miRNAs were expressed in all GEP-NETs grades (miR-10b-5p, miR-130b-3p, miR-192-5p, miR-194-5p, miR-210-3p, miR-214-3p, miR-7-5p, and miR-96-5p), but their expression level was different between differentiation grades. Among these, miR-96-5p were found to have increased expression levels from G1 to G3, and this may be probably related to the downregulation of FoxO1 gene by this miRNA (85).

The role of miRNAs as a diagnostic, prognostic, and chemoresistance tool was also explored in lung NENs. Recently, Yoshimoto et al. collected formalin-fixed paraffin-embedded samples of lung and GEP NETs, lung and GI adenocarcinomas, olfactory neuroblastomas, schwannomas, and related normal tissue for the analysis of their miRNA expression. After a very complex hierarchical clustering analysis, they found that lung and GI-NETs had a similar pattern of miRNA expression, suggesting a common origin between them, which was different from adenocarcinomas, SCLCs, and normal tissue. They also showed a distinct miRNA expression profile of SCLCs from lung carcinoids (89), and this may be useful to distinguish between low- and high-grade lung NENs. In addition, Rapa et al. showed that lung carcinoids have distinct miRNA expression profiles as compared with high-grade NECs, explaining that specific miRNAs might have potential implications as diagnostic tools or clinical biomarkers (102). As described for GEP-NENs, specific miRNA expression may also be used as prognostic markers (88). Specifically, overexpression of miR-92a2* and miR-7 and low levels of miR-150, miR-886-3p, miR-192, miR-200c, and miR-205 were described to be correlated to OS and PFS of SCLCs. MiR-92a2* and miR-7, along with mir-147 and miR-574-5p, were found to be associated with chemoresistance too (88, 90). A correlation with survival was also observed in typical and atypical carcinoids and LCNECs with upregulation of mir let-7d, miR-19, miR576-5p, miR-340*, and miR-1286, while overexpression miR-21 and low levels of miR-409-3p, miR-409-5p, and miR-431-5p correlated with the presence of lymph node metastases (88).

## Conclusions

Emerging evidence suggests an important role for biomarker identification and also NENs, in particular those with high-grade features. High-grade NENs can express different biomarkers (PD-L1, H-MSI status, miRNA expression patterns, and other alterations). A comprehensive exploration of biomarkers is still lacking as well as a molecular-driven clinical trial involving patients with NENs apart from the phase I/II multi-disease trial (**Table 2**). So considering that many of those biomarkers can be the target for new generations of drugs, with a subsequent significant clinical benefit, greater effort should be focused on spreading routine molecular analysis also in this setting of patients, like what usually happens with other malignancies. This may be important firstly for the patients themselves, giving the chance to obtain additional treatments with expanded access programs or nominal use, and secondly, because it may the basis for future clinical trials specific for this group of patients that may significantly change the currently untailored chemotherapy-based treatment strategies.

## Author Contributions

Conceptualization: MP, MMi, and SP. Methodology: MP, FM, NP, MN, and SC. Investigation: MP, AM, AR, GG, and FC. Resources: GR, FP, EC, and MMa. Data curation: MP, AM, AR, and FC. Writing—original draft preparation: MP, AM, AR, and FC. Writing—review and editing: MP, FM, NP, MN, SC, SP, GC, GS, AM, GC, and MB. Visualization: GR, FP, EC, and MMa. Supervision: MB, FB, MMi, and SP. All authors have read and agreed to the published version of the manuscript.

## Funding

The study was supported by the Italian Ministry of Health (ERP-2017-23671129 “PMTR-pNET”) to MM and by Fondazione IRCCS Istituto Nazionale Tumori Milano 5x1000 Funds—grant “Integrative molecular analysis of pure and combined lung large cell neuroendocrine carcinoma (LCNEC)” to MM. GC was supported by a FIRC-AIRC fellowship for Italy.

## Conflict of Interest

The authors declare that the research was conducted in the absence of any commercial or financial relationships that could be construed as a potential conflict of interest.

## Publisher’s Note

All claims expressed in this article are solely those of the authors and do not necessarily represent those of their affiliated organizations, or those of the publisher, the editors and the reviewers. Any product that may be evaluated in this article, or claim that may be made by its manufacturer, is not guaranteed or endorsed by the publisher.
